# Downregulation of miR-205 Modulates Cell Susceptibility to Oxidative and Endoplasmic Reticulum Stresses in Renal Tubular Cells

**DOI:** 10.1371/journal.pone.0041462

**Published:** 2012-07-30

**Authors:** Shiyo Muratsu-Ikeda, Masaomi Nangaku, Yoichiro Ikeda, Tetsuhiro Tanaka, Takehiko Wada, Reiko Inagi

**Affiliations:** Division of Nephrology and Endocrinology, University of Tokyo School of Medicine, Tokyo, Japan; North Carolina State University, United States of America

## Abstract

**Background:**

Oxidative stress and endoplasmic reticulum (ER) stress play a crucial role in tubular damage in both acute kidney injury (AKI) and chronic kidney disease (CKD). While the pathophysiological contribution of microRNAs (miRNA) to renal damage has also been highlighted, the effect of miRNA on renal damage under oxidative and ER stresses conditions remains elusive.

**Methods:**

We assessed changes in miRNA expression in the cultured renal tubular cell line HK-2 under hypoxia-reoxygenation-induced oxidative stress or ER stress using miRNA microarray assay and real-time RT-PCR. The pathophysiological effect of miRNA was evaluated by cell survival rate, intracellular reactive oxygen species (ROS) level, and anti-oxidant enzyme expression in miRNA-inhibited HK-2 or miRNA-overexpressed HK-2 under these stress conditions. The target gene of miRNA was identified by 3′-UTR-luciferase assay.

**Results:**

We identified 8 and 10 miRNAs whose expression was significantly altered by oxidative and ER stresses, respectively. Among these, expression of miR-205 was markedly decreased in both stress conditions. Functional analysis revealed that decreased miR-205 led to an increase in cell susceptibility to oxidative and ER stresses, and that this increase was associated with the induction of intracellular ROS and suppression of anti-oxidant enzymes. While increased miR-205 by itself made no change in cell growth or morphology, cell viability under oxidative or ER stress conditions was partially restored. Further, miR-205 bound to the 3′-UTR of the prolyl hydroxylase 1 (PHD1/EGLN2) gene and suppressed the transcription level of EGLN2, which modulates both intracellular ROS level and ER stress state.

**Conclusions:**

miR-205 serves a protective role against both oxidative and ER stresses via the suppression of EGLN2 and subsequent decrease in intracellular ROS. miR-205 may represent a novel therapeutic target in AKI and CKD associated with oxidative or ER stress in tubules.

## Introduction

Tubular cells are the main target of acute kidney injury (AKI). Reflecting the tubular cell damage, renal function is rapidly aggravated unless renal hypoperfusion or other causes of acute injury are promptly removed during the early, potentially reversible stage. Once renal damage reaches a certain threshold, the progression of renal disease is irreversible, consistent, and largely independent of the initial insult. The final common pathway in this process has been studied closely, and careful pathological analysis has revealed a good correlation between the degree of impairment of renal function in CKD and the extent of tubulointerstitial damage, indicating that tubular damage mediates the progression of chronic kidney disease (CKD). Among various insults that damage tubular cells in both AKI and CKD, recent studies have emphasized a role for pathogenic crosstalk between oxidative stress and endoplasmic reticulum (ER) stress.

Oxidative stress plays a crucial role in the damage induced by ischemia-reperfusion or hypoxia-reoxygenation. Oxidative stress damages endogenous kidney cells and decreases bioavailable nitric oxide, leading to vasoconstriction and the uncoupling of mitochondrial respiration with subsequent hypoxia of the kidney. Because oxygen consumption in hypoxic conditions increase oxidative stress [1], [2], hypoxia and oxidative stress act together to form in effect a vicious cycle. Hypoxia associated with oxidative stress in the kidney is thus both a central player in AKI as well as the final common pathway of CKD leading to end-stage kidney disease [3], [4].

**Table 1:** PCR primers used for detection of miRNAs.

miRNA	Product name	Catalog No.
U6	Hs_RNU6B_2 miScript Primer Assay	MS14000
miR-205	Hs_miR205_1 miScript Primer Assay	MS3780
miR-26a	Hs_miR-26a_2 miScript Primer Assay	MS29239
miR-29a	Hs_miR-29a_1 miScript Primer Assay	MS3262
miR-29b	Hs_miR-29b_1 miScript Primer Assay	MS6566
Pre-miR-205	Hs_mir-205_1_PR miScript Precursor Assay	MP1442

The ER maintains protein homeostasis via regulation of the biosynthesis, folding, trafficking, and modification of client proteins. These functions can be disturbed by various factors, such as hypoxia or starvation, and subsequently increases the accumulation of malfolded proteins in the ER, affects ER integrity (referred to as ER stress), and induces an intracellular stress signal, named the unfolded protein response (UPR). The UPR is classified into adaptive and maladaptive UPRs: the adaptive arm of the UPR attenuates ER stress and ensuring cell survival by increasing the expression of UPR target genes, including ER chaperones such as glucose-regulated protein 78 (GRP78), translation factors, and oxidant-detoxifying enzymes such as hemoxygenase 1 (HO-1), which both promote proper protein folding and attenuate protein translation. However, when ER stress is severe or prolonged and the adaptive UPR is insufficient to maintain ER homeostasis, the cell eventually triggers the maladaptive, proapoptotic UPR through multiple pathways mediated by CCAAT/enhancer-binding protein homologous protein (CHOP), JNK, caspase 12.

**Table 2:** PCR primers used for detection of mRNAs.

**human EGLN2 (prolyl hydroxylase domain-1:PHD1)**
forward 5′- GGC GAT CCC GCC GCG C - 3′
reverse 5′- CCT GGG TAA CAC GCC - 3′
**human EGLN1 (prolyl hydroxylase domain-2:PHD2)**
forward 5′- GCA CGA CAC CGG GAA GTT - 3′
reverse 5′- CCA GCT TCC CGT TAC AGT - 3′
**human EGLN3 (prolyl hydroxylase domain-3:PHD3)**
forward 5′- ATC GAC AGG CTG GTC CTC TA - 3′
reverse 5′- CTT GGC ATC CCA ATT CTT GT - 3′
**human 18S rRNA**
forward 5′- AAC GAA CGA GAC TCT GGC ATG - 3′
reverse 5′- CGG ACA TCT AAG GGC ATC ACA - 3′
**human glucose-regulated protein 78 (GRP78)**
forward 5′- GCC TGT ATT TCT AGA CCT GCC - 3′
reverse 5′- TTC ATC TTG CCA GCC AGC CAG TTG - 3′
**human copper/zinc superoxide dismutase (SOD1)**
forward 5′- AGG GCA TCA TCA ATT TCG AG - 3′
reverse 5′- TGC CTC TCT TCA TCC TTT GG - 3′
**human manganese superoxide dismutase (SOD2)**
forward 5′- GGA AGC CAT CAA ACG TGA CT - 3′
reverse 5′- CTG ATT TGG ACA AGC AGC AA - 3′
**human catalase**
forward 5′- TTT CCC AGG AAG ATC CTG AC - 3′
reverse 5′- ACC TTG GTG AGA TCG AAT GG - 3′
**human hemoxygenase 1 (HO-1)**
forward 5′- CGG GCC AGC AAC AAA GTG –3′
reverse 5′- AGT GTA AGG ACC CAT CGG AGA A - 3′
**human vascular endothelial growth factor (VEGF)**
forward 5′- TCT GAG CAA GGC CCA CAG GGA - 3′
reverse 5′- CCC TGA TGA GAT CGA GTA CAT CTT - 3′
**human glucose transporter 1 (GLUT1)**
forward 5′– CTT CAC TGT CGT GTC GCT GT –3′
reverse 5′- CCA GGA CCC ACT TCA AAG AA- 3′

Recent studies, including ours, have demonstrated that the adaptive UPR acts in various kidney cells to maintain ER integrity and that the excessive UPR state, namely dominant activation of the proapoptotic UPR, contributes to the development and progression of kidney diseases, including AKI and CKD [5]–[13]. Furthermore, recent evidence has demonstrated that the oxidative stress response enhances the UPR and vice versa. ROS produced by oxidative stress interfere with not only cellular redox-dependent reactions but also protein-folding capacity ultimately resulting in protein misfolding in the ER. Previous studies utilizing overexpression of anti-oxidant enzymes have emphasized the linkage of oxidative stress to the UPR. The ischemia-induced UPR was markedly less pronounced in rats overexpressing copper/zinc superoxide dismutase (SOD1), suggesting that superoxide radicals play a role in this pathological process [14]. Further, cadmium caused generation of ROS with subsequent induction of ER stress in a cultured porcine renal proximal tubular cell line, which in turn led to apoptosis; this cadmium-induced ER stress and apoptosis were significantly attenuated by transfection with manganese superoxide dismutase (SOD2) [15]. Paradoxically, ER stress also increases intracellular ROS production: increased protein disulfide bonding enhances ROS production in the ER lumen, and alteration of ER Ca^2+^ homeostasis increases cytosolic Ca^2+^, thereby stimulating mitochondrial ROS production. Thus, both oxidative stress and ER stress orchestrate tubular dysfunction. However, the mechanism of this effect remains unknown.

MicroRNAs (miRNAs) are short, endogenous, single-stranded ribonucleic acid (RNA) molecules that play a role as post-transcriptional regulators. MiRNAs are on average only 22 nucleotides long and are found in all eukaryotic cells. MiRNAs bind to complementary sequences on the 3′ untranslated region (UTR) of target messenger RNA transcripts (mRNAs), usually resulting in translational silencing, either by cleaving and degrading the target mRNA or blocking ribosomal translation of the targeted mRNA [16], [17]. Current knowledge suggests that two subsequent cleavages within the nucleus by the ribonuclease III family enzymes Drosha and Dicer result in the formation of mature miRNAs. After transcription of the primary miRNAs (pri-miRNAs), Drosha and the RNA-binding protein Pasha (also known as DGCR8) complex is processed into a hairpin structure, formally termed the precursor miRNA [18]. The precursor miRNA is transported into the cytoplasm, where it is cleaved by Dicer, and processed into a double-stranded product consisting of 22 nucleotides, and unwound to be a mature miRNA. This mature miRNA is incorporated into the RNA-induced silencing complex (RISC), and the RISC-miRNA complex specifically targets mRNAs and leads to negative regulation of protein synthesis or mRNA degradation. Several hundred miRNAs regulate approximately 50% of the human genome and are involved in virtually all biological processes, including cellular proliferation, apoptosis and differentiation. Thus, miRNA deregulation often results in impaired cellular function and development of disease. Recent findings have suggested that miRNA might also play a pathophysiological role in the progression of kidney diseases [19].

**Figure 1:**
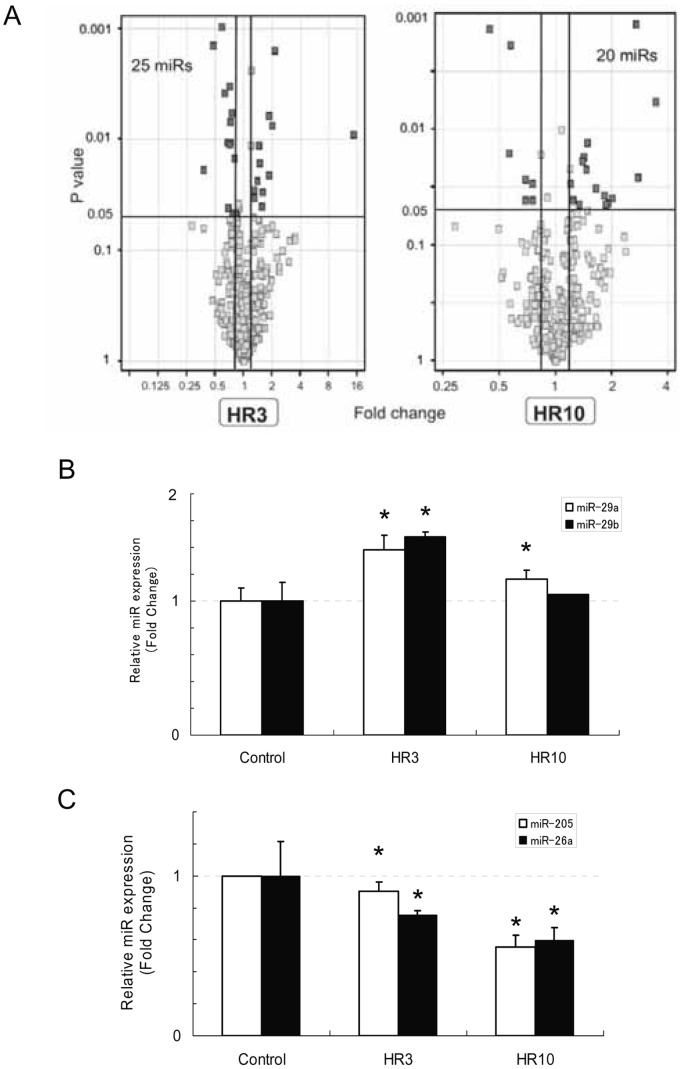
miRNA expression profiles under hypoxia-reoxygenation. (A) Volcano plots showing global changes in miRNA expression by hypoxia-reoxygenation (16 hours hypoxia followed by 3 hours (HR3, left figure) or 10 hours (HR10, right figure) reoxygenation). The horizontal dark line shows where p = 0.05 with points above the line having p<0.05 and those below it having p>0.05. Points with a fold-change of more than 1.2 or less than 0.8 with statistical significance are shown in dark gray. (B,C) The miRNA-specific quantitative real-time RT-qPCR analysis of commonly upregulated (B) or downregulated (C) miRNAs in HR3 and HR10. The expression patterns analyzed by qPCR were mostly consistent with those analyzed by microarray. The data represent the means ± S.E. of triplicate analysis from two independent experiments. *P<0.05 versus control of each miR group by standard *t*-test.

Among various pathogenesis, oxidative stress and ER stress have independently been reported as crucial inducers of tubular damage in both AKI and CKD, while recent studies have emphasized a role for pathogenic crosstalk between oxidative stress and ER stress. Although several findings have suggested that miRNA might play a pathophysiological role in the progression of kidney diseases, especially in connection with kidney fibrosis, the effect of miRNA on tubular damage under oxidative and ER stress conditions remains elusive. To elucidate whether these stress states or their crosstalk are regulated by miRNAs, we identified miRNAs that play a crucial role in tubular cell homeostasis under both oxidative and ER stress conditions. Moreover, we identified that PHD1/EGLN2 was a crucial protein in the crosstalk of oxidative stress and ER stress, and it was directly regulated by the miRNA. Interestingly, EGLN2 is not only well known to be a regulator of the stability of HIF, but recently its novel function that directly represses the ER stress regulator has also been reported [20].

## Materials and Methods

### Cell Culture

HK-2 cells possess morphologic characteristics of adult human proximal tubule epithelial cells [21]. The cells were maintained at 37°C in a 1∶1 mixture of Dulbecco’s Modified Eagle’s Medium (DMEM) and Ham’s F-12 Nutrient Mixture supplemented with 10% fetal bovine serum.

**Table 3:** miRNA expression change in HK-2 exposed to 3 hrs reoxygenation after hypoxia.

miRNA name	Fold Change (HR3/control)
hsa-miR-924	0.35
hsa-miR-205	0.47
kshv-miR-K12-3	0.50
hsa-miR-26a	0.53
hsa-miR-503	0.55
hsa-miR-21*	0.64
hsa-miR-135b	0.67
hsa-miR-769-5p	0.69
hsa-miR-30c-2*	0.71
hsa-miR-574-5p	0.77
hsa-miR-140-3p	0.78
hsa-miR-21	1.20
hsa-miR-301a	1.25
hsa-miR-151-5p	1.29
hsa-miR-155	1.33
hsa-miR-146a	1.37
hsa-miR-29a	1.38
hsa-let-7i	1.40
hsa-let-7e	1.41
hsa-miR-29b	1.45
hsa-miR-31*	1.48
hsa-miR-210	1.58
hsa-miR-7	1.71
hsa-miR-221*	2.09
hsa-miR-630	2.65

HR3∶ 16 hours hypoxia followed by 3 hours reoxygenation.

**Table 4:** miRNA expression change in HK-2 exposed to 10 hrs reoxygenation after hypoxia.

miRNA name	Fold Change (HR10/control)
hcmv-miR-UL148D	0.28
hsa-miR-205	0.37
hsa-miR-937	0.42
hsa-miR-181d	0.46
hsa-miR-32	0.47
hsa-miR-572	0.51
hsa-miR-378	0.53
hsa-miR-26a	0.68
hsa-miR-877*	0.69
hsa-miR-30c-2*	0.71
hsa-miR-563	0.72
hsa-miR-33a	0.75
hsa-miR-933	0.83
hsa-miR-221*	1.33
hsa-miR-29a	1.33
hsa-miR-29b	1.45
hsa-miR-7	1.46
hsa-miR-210	1.75
kshv-miR-K12-3	1.79
hsa-miR-630	2.27

HR10∶ 16 hours hypoxia followed by 10 hours reoxygenation.

### MiRNA Isolation and Quantitative Real-time RT-PCR

Total RNA, including mature miRNAs of cultured HK-2 was isolated utilizing an miRNeasy Mini kit (Qiagen, Hilden, Germany), and reverse-transcribed with an miScript Reverse Transcription kit (Qiagen) in accordance with the manufacturer’s instructions. cDNA was subjected to quantitative real-time RT-PCR using an miScript SYBR Green PCR kit (Qiagen) and iCycler PCR system (Bio-Rad, Hercules, CA). MiRNA-specific quantitative real-time RT-PCR was done using 3 ng of cDNA per reaction. The relative amounts of miRNAs and mRNAs were calculated with calibration by RNU6B and 18S rRNA, respectively. Each data represents the mean ± S.E. of three independent experiments performed in triplicate. The primers used are shown in Tables 1 and 2. All primers for miRNAs were purchased from Qiagen. The miRNA PCR products were cloned with T7 vectors utilizing a pGEM®-T Easy Vector System (Promega), and then sequenced to confirm sequence accuracy.

### Induction of Oxidative Stress and ER Stress Exposure

HK-2 cells for the hypoxia-reoxygenation groups were cultured in 0.1% O_2_ (Anaerocult A® sachets, Merck Chemicals, Darmstadt, Germany) for 16 hours and then reoxygenated for 3 hours (HR3) and 10 hours (HR10), respectively. For trypan blue exclusion assay, the cells were exposed to hypoxia for 16 hours followed by reoxygenation for 6 hours. The cells for ER stress groups were exposed to tunicamycin at 2 µg/ml (TUN) or thapsigargin at 0.5 µg/ml (THG) for 24 hours. Untreated cells were used as a control group. Oxidative stress and hypoxia were induced by exposure to 1000 µM hydrogen peroxide (H_2_O_2_) for 6 hours and 0.1% O_2_ for 16 hours without reoxygenation, respectively.

### MiRNA Microarray Analysis

HK-2 cells were plated in a 60-mm dish at a density of 5×10^4^ cells per plate. Cells for the hypoxia-reoxygenation and ER stress groups were treated as described above. Total RNA was then extracted, and RNA quality was assessed using the Agilent 2100 bioanalyzer system (Agilent Technologies, Santa Clara, CA). MiRNA expression profiles of the groups were compared using Human miRNA Microarray version 2 (Agilent Technologies), whose platform is capable of detecting 799 human miRNAs. Each group consisted of two assays.

The data obtained were analyzed with the GeneSpring GX software (Agilent Technologies). Uncorrected *P*-values for fold change data from microarrays were generated using one sample *t*-testing [22].

**Table 5:** miRNA expression change in HK-2 exposed to hypoxia-reoxygenation.

miRNA name	Fold Change (HR3/control)	Fold Change (HR10/control)
hsa-miR-205	0.47	0.37
kshv-miR-K12-3	0.50	1.79
hsa-miR-26a	0.53	0.68
hsa-miR-30c-2*	0.71	0.71
hsa-miR-29a	1.38	1.33
hsa-miR-29b	1.45	1.45
hsa-miR-210	1.58	1.75
hsa-miR-7	1.71	1.46
hsa-miR-221*	2.09	1.33
hsa-miR-630	2.65	2.27

HR3∶ 16 hours hypoxia followed by 3 hours reoxygenation.

HR10∶ 16 hours hypoxia followed by 10 hours reoxygenation.

### Transfection Procedures

miR-205 was knocked down or overexpressed by transfection with a locked nucleic acid-labeled miRNA inhibitor (miRCURY LNA™ microRNA Power Inhibitor, Exiqon, Vedbaek, Denmark) or miRNA mimic (miCENTURY OX Synthetic microRNA Collection, Cosmo Bio, Tokyo, Japan). Control inhibitor (miRCURY LNA™ microRNA Inhibitor Negative Controls, Exiqon) and control mimic (Non-target microRNA, Cosmo Bio) were used as a negative control, respectively. HK-2 cells were transfected with LNA-miR-205 inhibitor or control inhibitor with a HiPerFect Transfection Reagent kit from Qiagen in accordance with the manufacturer’s instructions. Successful transfection was confirmed using miRNA-specific quantitative real-time RT-PCR for miR-205.

### Cell Viability

Cell viability was assessed using trypan blue exclusion assay with a final dye concentration of 0.4% at 24 hours posttransfection with miR-205 modulators (inhibitor or mimic) or negative control. We also performed lactate dehydrogenase (LDH) release assay. LDH in the cultured medium and in the cell lysate were measured according to manufacturer’s instruction (Kainos Laboratories, Tokyo, Japan). Percentage of cell lysis was calculated from the amount of LDH in the medium divided by the total amount of LDH in the medium and cell lysate.

**Figure 2:**
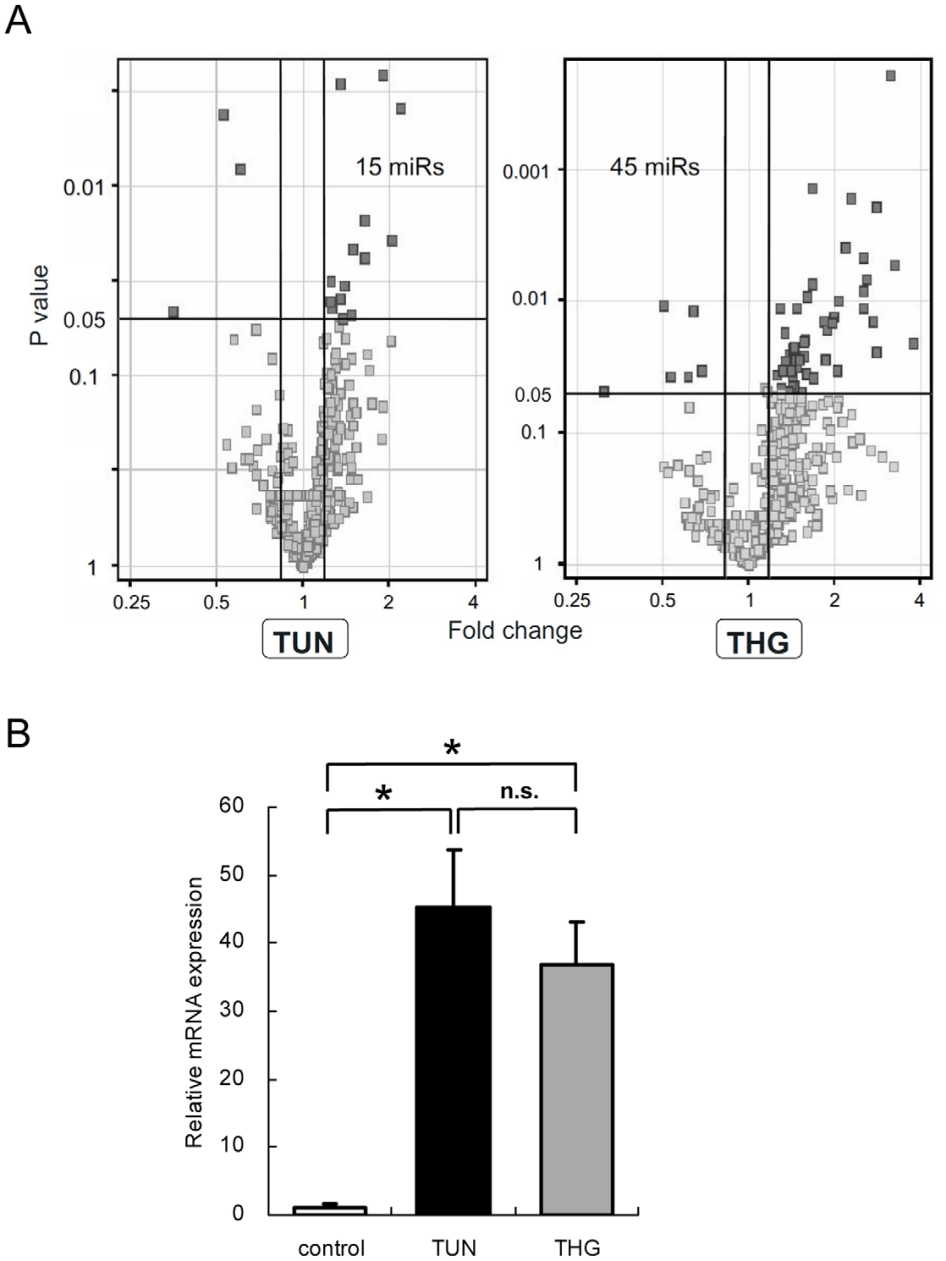
miRNA expression profiles under ER stress. (A) Volcano plots showing the global changes in miRNA expression by ER stress. Points with a fold-change more than 1.2 or less than 0.8 with statistical significance are shown in dark gray. (B) Real-time RT-qPCR analysis revealed equally increased expression of GRP78 in HK-2 exposed to TUN, or THG, indicating that ER stress induced by each chemical was equivalent. The data represent the means ± S.E. of triplicate analysis from two independent experiments. *P<0.05 by Tukey’s multiple comparison test.

### Flow Cytometry

Twenty-four hours posttransfection, HK-2 cells were treated with 10 µM of chloromethyl-2′,7′-dichlorofluorescein diacetate, acetyl ester (CM-H_2_DCFDA; Molecular Probes) for 30 min in the dark to detect intracellular ROS, challenged with H_2_O_2_ at 500 µM for 2 min, and trypsinized. Intracellular ROS production was analyzed using a FACScalibur (Becton Dickinson, Franklin Lakes, NJ) equipped with a 488-nm argon laser and the CELL Quest Pro software (Becton Dickinson). CM-H2DCFDA fluorescence was analyzed in the FL-1 (530 nm) channel. Forward and side scatter were used to gate out cellular fragments, and data were collected for 1×10^4^ cells per sample.

**Table 6:** miRNA expression change in HK-2 exposed to tunicamycin.

miRNA name	Fold Change (TUN/control)
hsa-miR-202	0.27
hsa-miR-483-5p	0.49
hsa-miR-205	0.53
hsa-miR-503	0.61
hsa-miR-542-5p	0.61
hsa-miR-584	0.63
hsa-miR-106a	0.67
hsa-miR-215	0.71
hsa-miR-33a	0.73
hsa-miR-188-5p	0.74
hsa-miR-133a	0.74
hsa-miR-99b	0.79
hsa-miR-17*	0.80
hsa-miR-181a-2*	0.80
hsa-miR-181a*	1.90

TUN: tunicamycin of 2 µg/ml for 24 hours.

**Table 7:** miRNA expression change in HK-2 exposed to thapsigargin.

miRNA name	Fold Change (THG/control)
hsa-miR-150*	0.003
hsa-miR-663	0.07
hsa-miR-134	0.09
hsa-miR-671-3p	0.15
hsa-miR-629*	0.17
hsa-miR-423-5p	0.18
hsa-miR-423-3p	0.20
hsa-miR-202	0.24
hsa-miR-182	0.24
hsa-miR-500*	0.29
kshv-miR-K12-3	0.32
hsa-miR-215	0.34
hsa-miR-431*	0.39
hsa-miR-205	0.39
hsa-miR-425	0.48
hsa-miR-572	0.50
hsa-miR-503	0.50
hsa-miR-133a	0.53
hsa-miR-371-5p	0.54
kshv-miR-K12-9*	0.59
hsa-miR-30c-2*	0.59
hsa-miR-125a-3p	0.60
hsa-miR-99b	0.62
hsa-miR-193b	0.62
hsa-miR-210	0.63
hsa-miR-660	0.63
hsa-miR-30a*	0.64
hsa-miR-532-5p	0.64
hsa-miR-93	0.65
hsa-miR-361-5p	0.66
hsa-miR-181b	0.68
hsa-miR-128	0.68
hsa-miR-24	0.70
hsa-miR-19b	0.70
hsa-miR-130a	0.70
hsa-miR-130b	0.71
hsa-miR-429	0.72
hsa-miR-181a-2*	0.74
hsa-miR-129-3p	0.75
hsa-miR-638	0.76
hsa-miR-574-5p	0.76
ebv-miR-BART13	0.78
hsa-miR-193a-3p	0.80
hsa-miR-181a*	1.56
ebv-miR-BART10*	2.02

THG: thapsigargin of 0.5 µg/ml for 24 hours.

### Western Blot Analysis

Nuclear and cytoplasmic fractions from HK-2 cells were prepared using NE-PER Nuclear and Cytoplasmic Extraction Reagents kit (Pierce, Rockford, IL) according to manufacturer’s instruction. Nuclear fraction was used in immunoblotting for HIF or ATF4, and cytoplasmic fraction was used for Nε-carboxyethyl lysine (CEL), HO-1, SOD1 and prolyl hydroxylase 1 (PHD1/EGLN2). Protein extracts were electrophoresed on 10–12% SDS-PAGE, and transferred to a polyvinylidene fluoride membrane (Amersham Biosciences, Piscataway, NJ). For detection of HO-1, SOD1, CEL, EGLN2, ATF4, HIF-1α and HIF-2α, membranes were incubated with anti-HO-1 antibody (1∶1000; Enzo Life Science, Farmingdale, NY), anti-SOD1 antibody (1∶200; Santa Cruz Biotechnology, Santa Cruz, CA), anti-CEL antibody (1∶200; TransGenic, Kobe, Japan), anti-EGLN2 antibody (1∶1000; EPITOMICS, Burlingame, CA), anti-ATF4 antibody (1∶200; Santa Cruz Biotechnology), anti-HIF-1α antibody (1∶1000; Novus Biologic, Litteleton, CO), anti-HIF-2α antibody (1∶1000; Novus Biologic) as the first antibody, respectively. Rabbit anti-actin polyclonal antibody (1∶5000; Sigma, St. Louis, MO) was used as the control. The ECL plus kit (Pierce) was used for detection. Density of bands was analyzed using Image J version 1.45 s software (National Institutes of Health, Bethesda, MD).

**Table 8:** miRNA expression change in HK-2 exposed to ER stress.

miRNA name	Fold Change (TUN/control)	Fold Change (THG/control)
hsa-miR-202	0.27	0.24
hsa-miR-205	0.53	0.39
hsa-miR-503	0.61	0.50
hsa-miR-215	0.71	0.34
hsa-miR-133a	0.74	0.53
hsa-miR-99b	0.79	0.62
hsa-miR-181a-2*	0.80	0.74
hsa-miR-181a*	1.90	1.56

TUN: tunicamycin of 2 µg/ml for 24 hours.

THG: thapsigargin of 0.5 µg/ml for 24 hours.

**Figure 3:**
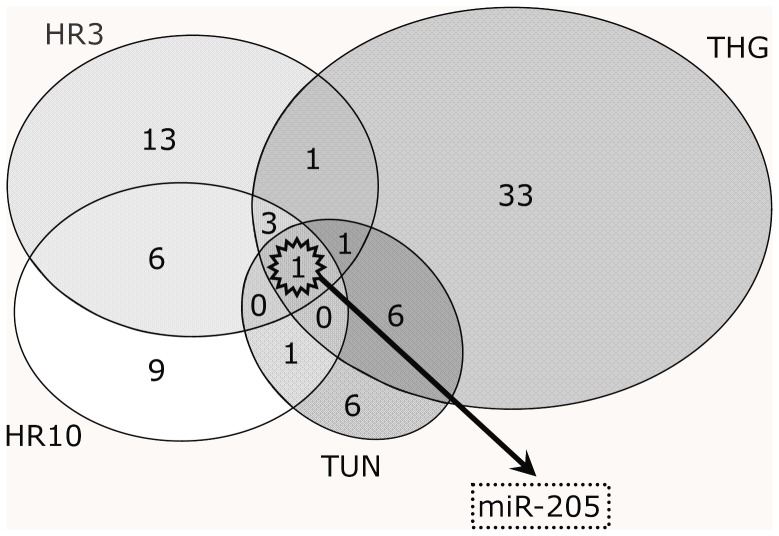
Venn diagram showing the result of merging miRNA expression profiles of oxidative stress induced by hypoxia-reoxygenation with those of ER stress. The expression of only one miRNA, miR-205, was consistently altered under both stress conditions.

### Mutagenesis

Using the primer design guidelines included in the QuickChange XL Site-Directed Mutagenesis Kit (Agilent Technoligies, Santa Clara, CA), we chose forward primer 5′-GGTCATGACCCCATTAGGTCCCGGCAGCTGGGAGGAGGC and reverse primer 5′-GCCTCCTCCCAGCTGCCGGGACCTAATGGGGTCATGACC. PCR was performed using the *Pfu Turbo* DNA Polymerase according to manufacturer’s instructions. The PCR product was then digested with Dpn I to remove template DNA, followed by transformation of XL10-Gold® Ultracompetent Cells. Cells were then plated on Kanamycin-containing Luria-Bertani (LB) agar overnight at 37°C. Individual colonies were then grown in LB/Kanamycin medium, and plasmid DNA was extracted using the PureYield™ Plasmid Midiprep System (Promega, Madison, WI). Purified DNA was then verified by sequencing.

**Figure 4:**
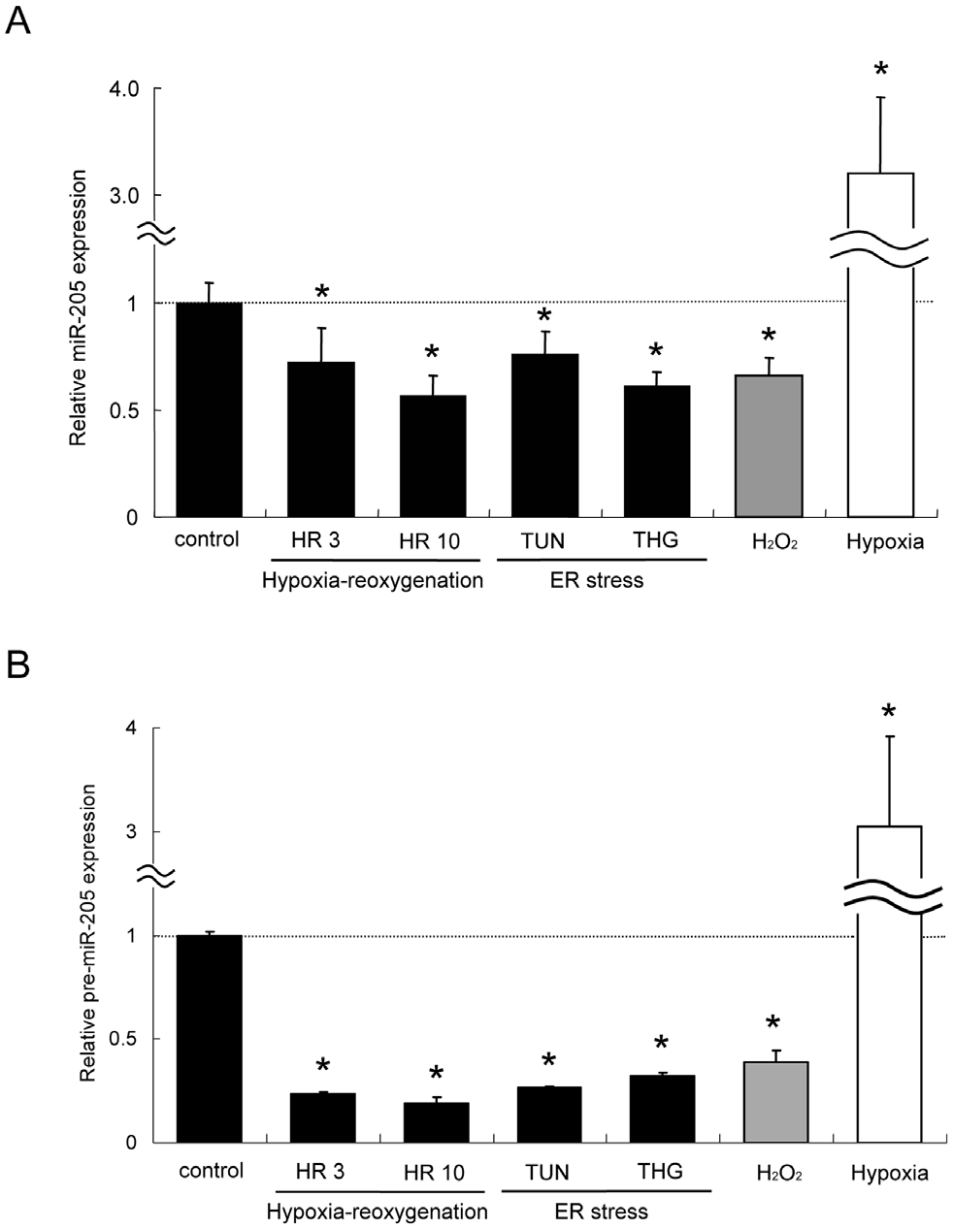
Expression changes in miR-205 by oxidative stress or ER stress. (A) Real-time RT-qPCR analysis revealed decreased expression of miR-205 in HK-2 exposed to hypoxia-reoxygenation, TUN, or THG. A decrease in miR-205 expression was also observed in HK-2 treated with H_2_O_2_. Meanwhile, hypoxia without reoxygenation (0.1% O_2_ for 16 hours) markedly increased miR-205 expression. The data are the means ± S.E. of triplicate analysis from three independent experiments. (B) Expression of a precursor of miR-205 (pre-miR-205) was also changed in a similar pattern to miR-205. *P<0.05 versus control by Dunnett’s multiple comparison test.

### Transient Transfection and Luciferase Assay

The EGLN2 3′-UTR miRNA target sequence expression clone in pEZX-MT01 vector with fLuc was purchased from GeneCopoeia (miTarget ™ miRNA 3′-UTR Target Clone, Rockville, MD). For reporter assays, HK-2 cells were transiently co-transfected with either wild type or mutant EGLN2 3′-UTR reporter plasmid and either miR-205 modulators (inhibitor or mimic) or negative control by utilizing a Lipofectamine 2000 (Invitrogen, Carlsbad, CA).

The hypoxia-responsive reporter vector with tandem copies of the hypoxia-responsive element (HRE) from the rat vascular endothelial growth factor gene subcloned into 5′ region of hmCMV-promoter-luciferase transcription unit (pHRE-luc) was constructed in our previous work [23]. Five-hundred nanogram of pHRE-luc, 25 ng of CMV-renilla luciferase (Promega) per 1×10^5^ HK-2 cells, and either miR-205 modulators (inhibitor or mimic) or negative control were co-transfected by utilizing a Lipofectamine 2000 (Invitrogen) in 24-well culture dishes.

**Figure 5:**
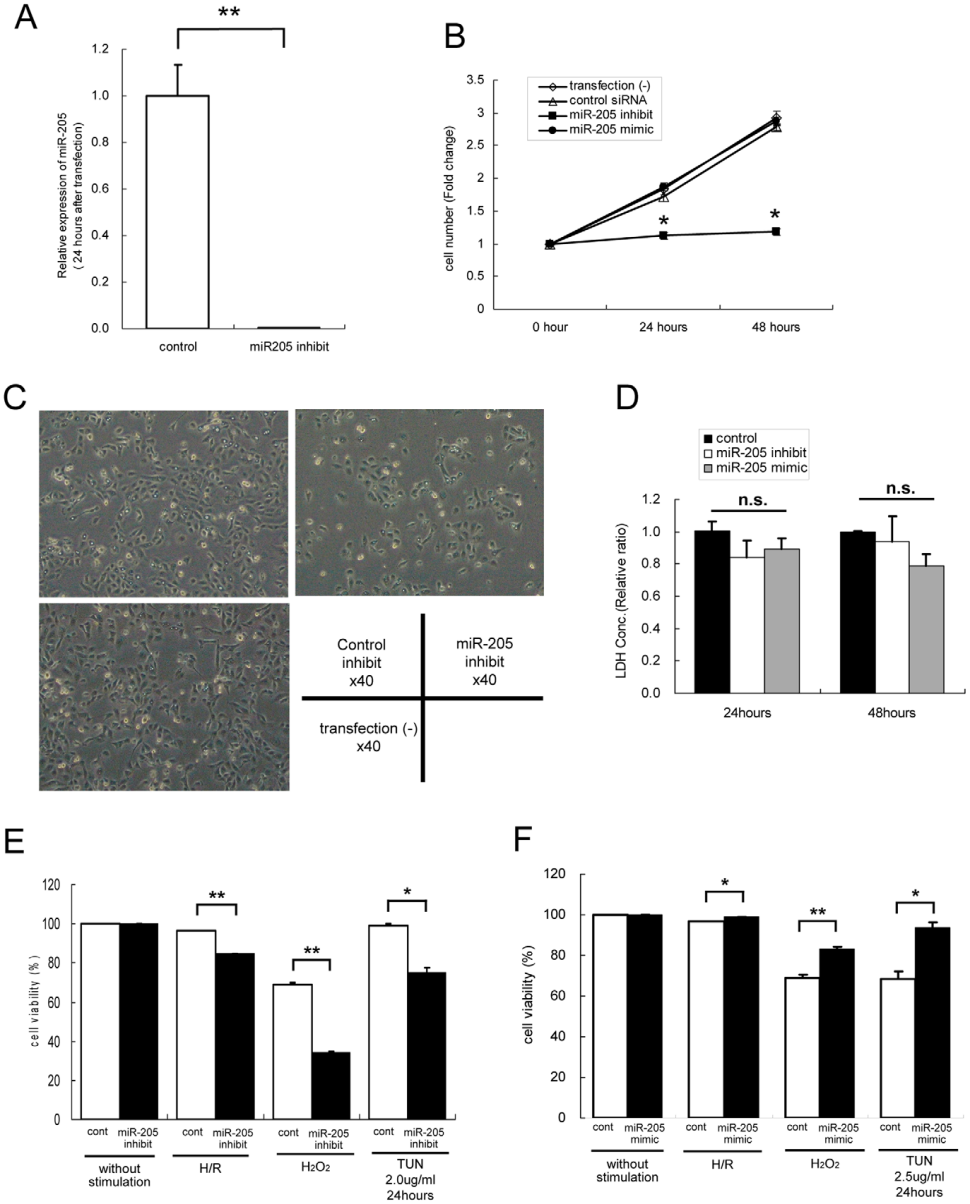
Change in cell survival under oxidative and ER stresses in miR-205-inhibited HK-2. (A) Inhibition of miR-205 in HK-2. Real-time RT-qPCR analysis revealed that transfection of an LNA-miR-205 inhibitor into HK-2 for 24 hours significantly inhibited miR-205 expression. The data represent the means ± S.E. of triplicate analysis from three independent experiments. **P<0.01 versus control siRNA transfected HK-2 by standard *t*-test. (B) In miR-205-inhibited HK-2, the cell number was significantly fewer at 24 and 48 hours after transfection than that of with HK-2 transfected with negative control. In contrast, up-regulation of miR-205 had no effects on the cell number. The data represent the means ± S.E. of triplicate counts from three independent experiments. *P<0.05 versus negative control transfected HK-2 at each time point by standard *t*-test. (C) Representative pictures of HK-2, 24 hours after transfection with miR-205 inhibitor or control inhibitor. Although cell growth was repressed, cell morphology of miR-205-inhibited HK-2 was not different from control siRNA transfected HK-2. (D) LDH release assay showed that modulation of miR-205 did not affect cell death, suggesting that the decrease in cell number in miR-205-inhibited HK-2 was not due to cell injury, but rather to repression of cell growth. The data represent the means ± S.E. of triplicate analysis from three independent experiments. Statistical analysises were performed by Tukey’s multiple comparison test. (E,F) Trypan blue exclusion assay showed the cell viability changes in miR-205 modulated HK-2 (▪) exposed to hypoxia-reoxygenation (0.1% O_2_ for 16 hours followed by 6 hours reoxygenation), H_2_O_2_ (1000 µM for 6 hours) or TUN (2 µg/ml for 24 hours) compared to HK-2 transfected with negative control (□). Down-regulation of miR-205 led to significant decrease in cell viability by each stimulation (E), wheares up-regulation of miR-205 vice versa (F). Cell viabilities were not changed without any stimulations. The data are the means ± S.E. of triplicate counts from three independent experiments. *P<0.05, **P<0.01 by standard *t*-test.

Part of transfected cells were exposed to oxidative stress or ER stress as follows before harvested by 100 µl of passive protein lysis buffer for dual luciferase assay. Oxidative stress was induced by 16 hours hypoxia followed by 8 hours reoxygenation or 1000 µM H_2_O_2_ for 3 hours. ER stress was induced by TUN at 1.5 µg/ml for 24 hours.

The effects of miR-205 on EGLN2 or HIF were estimated by firefly luciferase activity and measured with a Dual Luciferase Assay kit (Promega, Madison, WI) 24 hours after transfection, with the results normalized with *Renilla* luciferase. A Lumat 9507 luminometer (EG and Berthold, Bad Wildbad, Germany) was used for measurement.

### Statistical Analysis

All data are reported as the mean ± SEM of the indicated number of measurements. Significance levels between groups were calculated with either standard *t-*tests or Dunnett’s and Tukey’s multiple comparison tests. Differences with P<0.05 were considered statistically significant.

**Figure 6:**
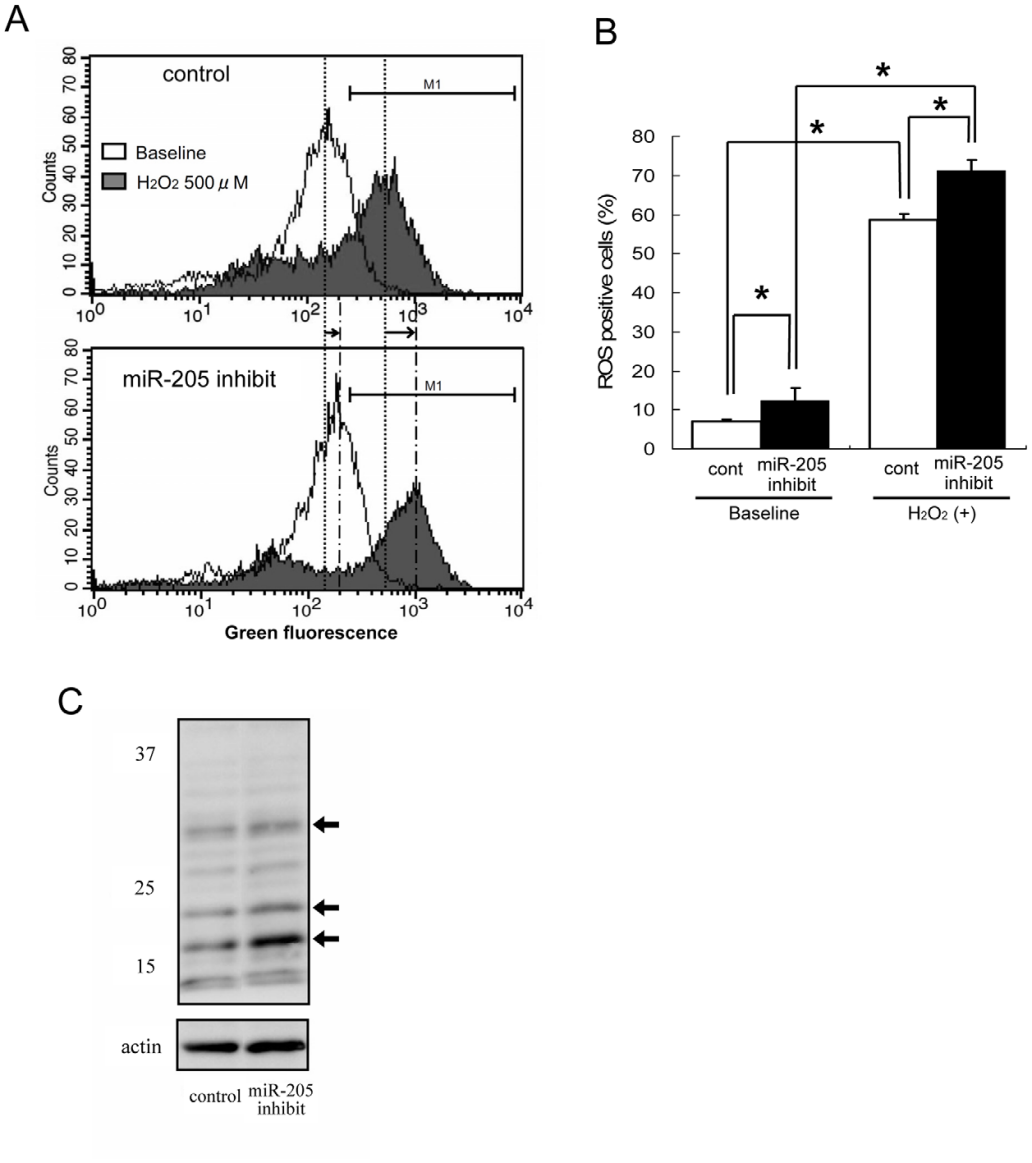
Suppression of miR-205 increased oxidative stress. (A) Representative figure of the result of FACS. Formation of intracellular ROS was confirmed using CM-H2DCFDA, a dye that emits green fluorescence on reaction with ROS. miR-205-inhibited HK-2 cells were exposed to H_2_O_2_ in a short time as an oxidative stress inducer. The fluorescence value of miR-205-inhibited HK-2 was higher than the control both before (uncolored area) and after (gray area) induction by H_2_O_2_. ROS-positive expression boundaries are marked by horizontal lines (M1) on the histogram plots. (B) Changes in the proportion of ROS-positive cells by miR-205 inhibition before and after H_2_O_2_ treatment. Intracellular ROS was significantly increased in miR-205-inhibited HK-2 (▪) compared to negative control transfected HK-2 (□) even before the cells were exposed to H_2_O_2_. The data represent the means ± S.E. of duplicate analysis from three independent experiments. *P<0.05 by Tukey’s multiple comparison test. (C) Western blot analysis of CEL showed that the intensity of bands with a molecular weight corresponding to 32, 23, and 17 kDa (black arrows) were increased in miR-205-inhibited HK-2 compared with control inhibitor transfected HK-2, suggesting that intracellular ROS was increased by down-regulation of miR-205.

## Results

### MiRNA Expression Profile in HK-2 Under Oxidative Stress

MiRNA expression profiles under oxidative stress were evaluated by assessing changes in miRNA expression by miRNA microarray analysis in a human proximal tubular cell line, HK-2, exposed to hypoxia-reoxygenation. In this analysis, the cells were exposed to hypoxia for 16 hours followed by reoxygenation for 3 (HR3) or 10 hours (HR10). A perspective on the global changes in miRNA expression is presented by volcano plots in Figure 1A. We selected miRNAs whose expression changes were increased more than 1.2 times or decreased less than 0.8 times, with statistical significance. Of the 799 miRNAs we tested, the expression levels of 25 and 20 miRNAs were significantly altered in the HR3 and HR10 groups, respectively, as compared to those in untreated cells (Table 3 and 4). Among these, 10 miRNAs showed a continuous change in expression during the experimental period (Table 5).

**Figure 7:**
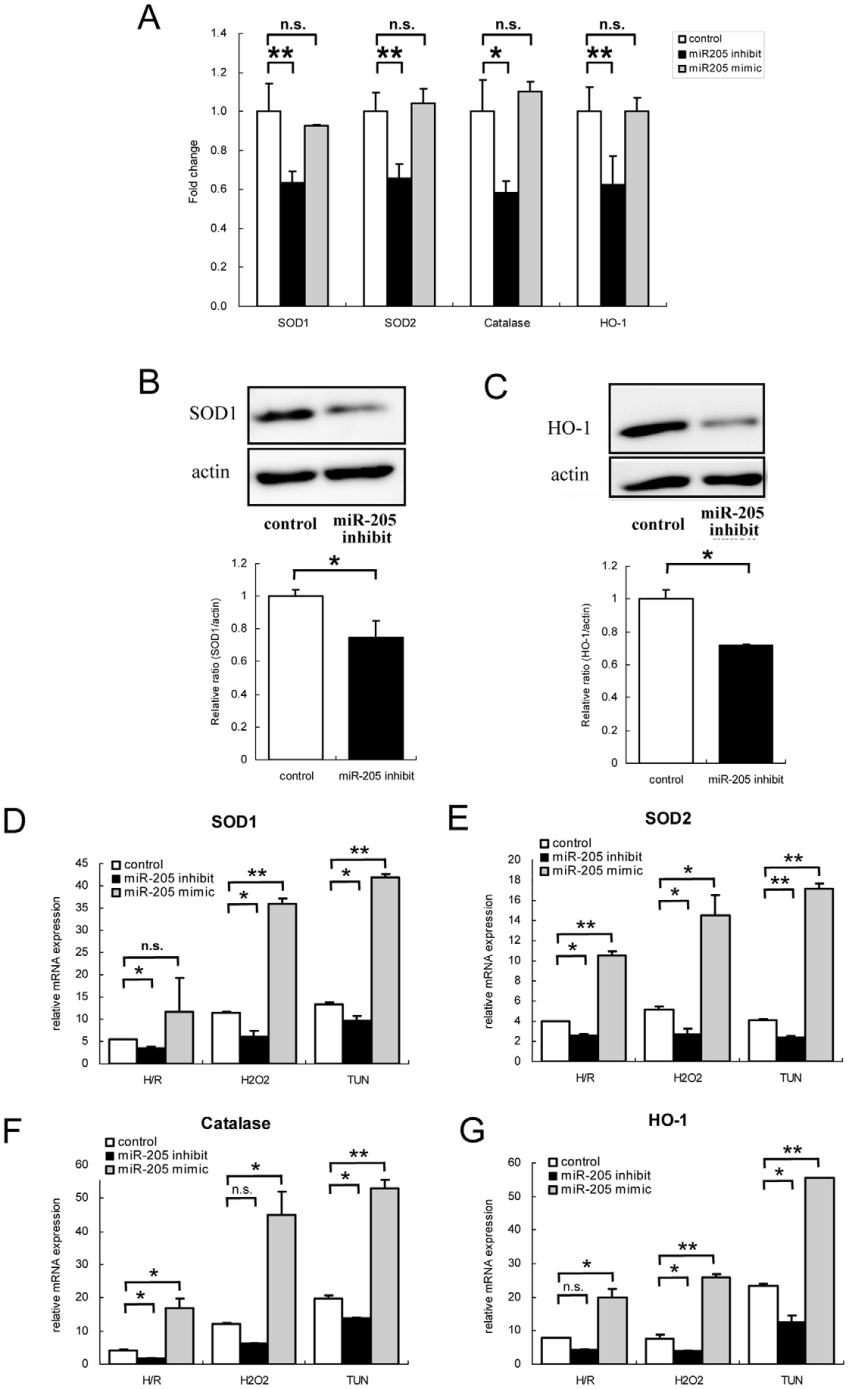
Decreased expression of anti-oxidant enzymes by miR-205 inhibition. (A) Quantitative real-time RT-PCR analysis revealed decreased expression of anti-oxidant enzymes in miR-205-inhibited HK-2. Baseline expression levels of antioxidant enzymes SOD1, SOD2, catalase, and HO-1 were significantly lower in miR-205-inhibited HK-2, but not significantly changed in miR-205 overexpressed HK-2 compared to negative control transfected HK-2. The data represent the means ± S.E. of duplicate analysis from three independent experiments.*P<0.05, **P<0.01 by Dunnett’s multiple comparison test. (B,C) Western blot analysis of cytoplasmic extracts from miR-205-inhibited HK-2 or negative control transfected HK-2 was performed to detect SOD1 (B) or HO-1 (C). Both SOD1 and HO-1 were also decreased at protein level by down-regulation of miR-205. Th results of densitometry represent the means ± S.E. of triplicate analysis from two independent experiments.*P<0.05 by standard *t*-test. (D-G) Quantitative real-time RT-PCR analysis revealed that stress-induced increases of SOD1, SOD2, Catalase and HO-1 were enhanced by up-regulation of miR-205, indicating that cell protective effect of miR-205 was excerted via modulation of these anti-oxidant enzymes. The data represent the means ± S.E. of triplicate analysis from two independent experiments.*P<0.05, **P<0.01 by standard *t*-test.

To confirm the results of miRNA microarray analysis, we performed quantitative real-time RT-PCR followed by sequencing analysis of the PCR products. We selected two miRNAs with the highest basal expression levels for each of the up-regulated and down-regulated miRNA groups. Changes in the expression of these four miRNAs (miR-26a, miR-29a, miR-29b, miR-205) analyzed by real-time RT-PCR were consistent with those by miRNA microarray (Figure 1B and 1C), although the change in miR-29b at HR10 was diminished.

### MiRNA Expression Profile in HK-2 Under ER Stress

In this study, HK-2 cells were exposed to the ER stress inducers, tunicamycin at 2 µg/ml (TUN) or thapsigargin at 0.5 µg/ml (THG) for 24 hours. Volcano plot displays of changes in miRNAs by these ER stress inducers are shown in Figure 2A. Though the mechanisms for triggering ER stress by TUN and THG are different, the expression levels of GRP 78, a central regulator of the UPR, were equivalently increased by both ER stress inducers, indicating that these miRNA profiles really reflect ER stress rather than an off-target effect of these chemicals (Figure 2B).

When we selected altered miRNAs based on maximum expression signal intensity in the same manner as in the HR-induced oxidative stress group, expression levels of 15 and 45 miRNAs were significantly changed in the TUN and THG groups, respectively (Table 6 and 7).

**Figure 8:**
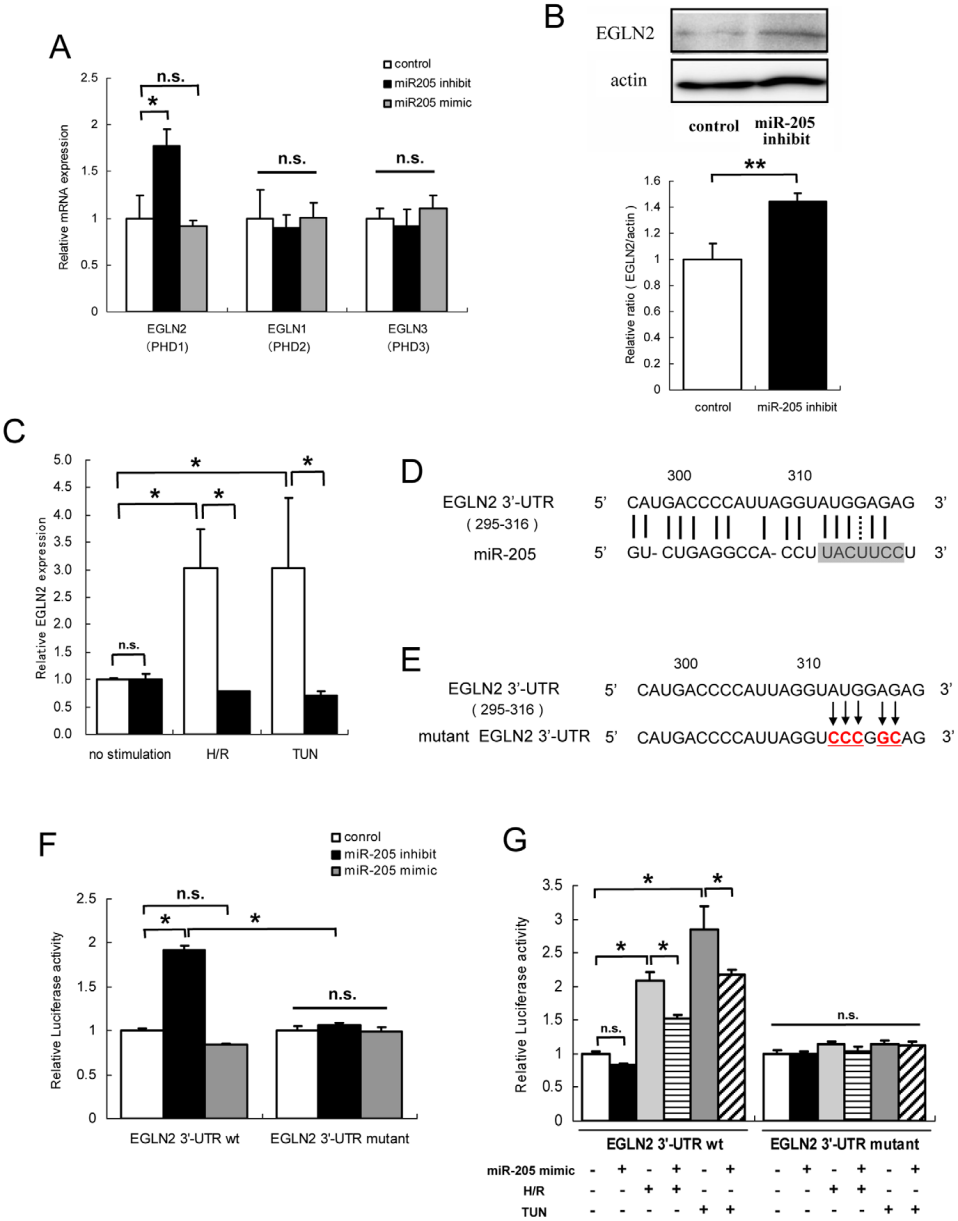
EGLN2 as a novel downstream target of miR-205. (A) Quantitative real-time RT-PCR analysis revealed that the expression of EGLN2 was increased by miR-205 inhibition, but not significantly altered by overexpression of miR-205 compared to negative control transfected HK-2. No changes were seen in expression levels of EGLN1 (PHD2) or EGLN3 (PHD3). The data represent the means ± S.E. of triplicate analysis from three independent experiments. *P<0.05 by Dunnett’s multiple comparison test. (B) Western blot analysis showed that EGLN2 was also increased at protein level by miR-205 inhibition. The result of densitmetry represent the means ± S.E. of triplicate analysis from two independent experiments.**P<0.01 by standard *t*-test. (C) Quantitative real-time RT-PCR analysis revealed that the expression level of EGLN2 was increased in HK-2 exposed to hypoxia-reoxygenation or TUN, but these increases were supressed by miR-205 overexpression (▪) compared to negative control transfected HK-2 (□). *P<0.05 by Tukey’s multiple comparison test. (D) EGLN2 (transcript variant 1) contains the predicted binding site for miR-205 in its 3′-UTR. The predicted pairing of the mRNA target region (top) and miRNA (bottom) is as indicated, wherein solids line indicates hydrogen base pairing and the dotted line indicates Watson-Crick/wobble base pairing. The shaded sequence indicates the seed region of miR-205. (E) Mutant 3′-UTR sequence that abolished binding to miR-205. (F) HK-2 cells were transiently cotransfected with a luciferase reporters expressing EGLN2 3′-UTR or mutant EGLN2 3′-UTR miRNA target sequence and miR-205 mimic, miR-205 inhibitor, or negative control. At 24 hours posttransfection, firefly luciferase activities were measured and normalized with *Renilla* luciferase activities. Relative luciferase activity was increased by miR-205 inhibition when compared with control, while mutant EGLN2 3′-UTR had no effect on luciferase activity, indicating that miR-205 acts on the 3′-UTR of the EGLN2. Though transfection of HK-2 with EGLN2 3′-UTR vector along with miR-205 overexpression tended to decrease luciferase activity, this change was not sicnificant. The data represent the means ± S.E. of triplicate measurements from two independent experiments. *P<0.05 by Tukey’s multiple comparison test. (G) When transiently transfected HK-2 with EGLN2 3′-UTR vector were exposed to oxidative or ER stress, the relative luciferase activities were significantly increased, and these increases were canceled with mutant EGLN2 3′-UTR vectors. Furthermore, co-transfection of EGLN2 3′-UTR vector with miR-205 mimic partially restrained these stress-induced increases of luciferase activities. The data represent the means ± S.E. of triplicate measurements from two independent experiments. *P<0.05 by Tukey’s multiple comparison test.

Among these we detected eight miRNAs, which showed significant changes in expression by both TUN and THG (Table 8). The degree of expression change in the two groups tended to be the same for all eight miRNAs. To confirm these findings, we performed quantitative real-time RT-PCR followed by sequencing analysis of the PCR products. Among eight miRNAs we confirmed the down-regulation of miR-205 expression by both TUN and THG (Figure 3 and 4A). The change of the other miRNAs was undetectable by quantitative real-time RT-PCR. It might be due to sensitivity of PCR or relatively low level of basal expression.

### Identification of miRNA Altered by Both Oxidative Stress and ER Stress

Merging miRNA expression profiles of oxidative stress induced by hypoxia-reoxygenation with those of ER stress identified one miRNA, miR-205, whose expression was markedly decreased under both stress conditions (Figure 3). According to the result of this microarray analysis, miR-205 expression level in HK-2 at baseline was equivalent to other well-studied miRNAs which are related to hypoxia or kidney diseases, such as miR-210 [24]–[26], miR-29a [27], miR-200a and miR-200b [28] (relative ratio of basal expression level to miR-205 was 1.25, 7.8, 0.86, 3.86, respectively).

The change in miR-205 expression in HK-2 under various stress conditions was confirmed by quantitative real-time RT-PCR followed by sequencing analysis (fold change compared to control of 0.72±0.16 in HR3 (P<0.05), 0.56±0.10 in HR10 (P<0.01), 0.76±0.11 in TUN (P<0.05), 0.61±0.07 in THG (P<0.01)) (Figure 4A). A decrease in miR-205 expression was also observed in HK-2 under H_2_O_2_-induced oxidative stress (fold change compared to control of 0.66±0.08, P<0.05) (Figure 4A). In contrast, hypoxia *per se* significantly increased miR-205 expression (fold change compared to control of 3.61±0.79, P<0.01) (Figure 4A). This change in miR-205 expression was supported by the finding of a similar pattern of change in the expression of pre-miR-205, a precursor of miR-205 (fold change compared to control of 0.24±0.01 in HR3 (P<0.01), 0.19±0.03 in HR10 (P<0.01), 0.26±0.005 in TUN (P<0.05), 0.32±0.02 in THG (P<0.05), 0.39±0.05 in H_2_O_2_ (P<0.01), and 3.16±0.91 in hypoxia (P<0.05)) (Figure 4B). These results suggest that miR-205 is generated through the proper processes and the expression changes of miR-205 by oxidative stress and ER stress in this experiment are reliable.

**Figure 9:**
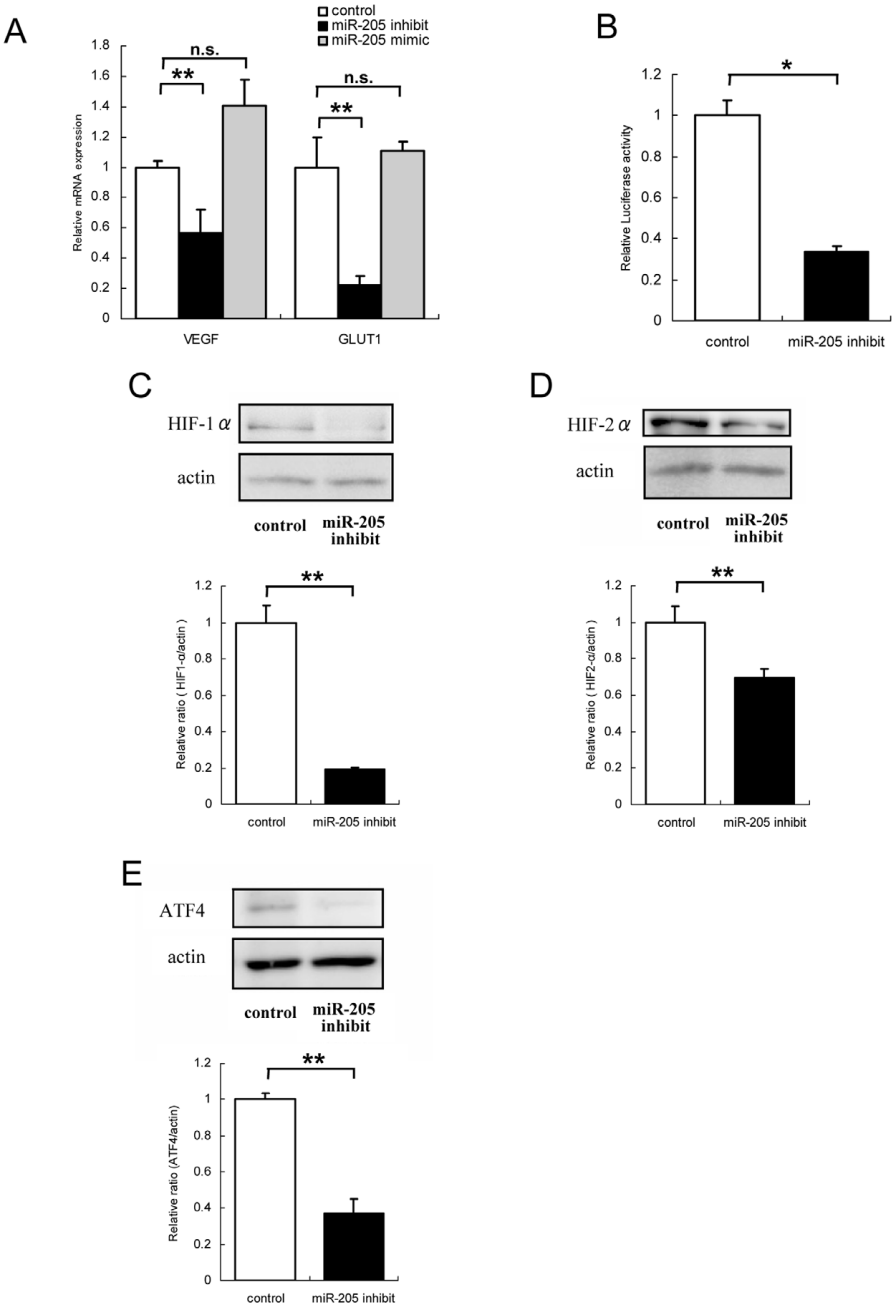
Expression changes of HIF and ATF4 modulated by miR-205. (A) Quantitative real-time RT-PCR analysis revealed that the expression level of both VEGF and GLUT1, the down stream targets of HIF, were decreased by miR-205 down-regulation, but not significantly changed by miR-205 up-regulation. The data represent the means ± S.E. of triplicate measurements from two independent analysis. **P<0.01 by Dunnett’s multiple comparison test. (B) HK-2 cells were transiently cotransfected with a HRE-luciferase reporter and miR-205 inhibitor or negative control. At 24 hours posttransfection, firefly luciferase activities were mesured and normalized with *Renilla* luciferase activities. Relative luciferase activity was significantly decreased by miR-205 inhibition, suggesting that HIF was indirectly regulated by miR-205. The data represent the means ± S.E. of triplicate measurements from two independent analysis. *P<0.05 by standard *t*-test. (C, D) Western blot analysis of HIF-1α (C) and HIF-2α (D) revealed that both HIF proteins were significantly decreased by down-regulation of miR-205. The results of densitometry represent the means ± S.E. of triplicate measurements from two independent analysis. **P<0.01 by standard *t*-test. (E) Western blot analysis of ATF4 revealed that down-regulation of miR-205 led to significant decrease in nuclear ATF4 accumulation, indicating that ATF4 was also indirectly regulated by miR-205. The results of densitometry represent the means ± S.E. of triplicate measurements from two independent analysis. **P<0.01 by standard *t*-test.

### Modulation of miR-205 Expression Influenced Cell Susceptibility to Oxidative Stress or ER Stress

The biological effects of the down-regulation of miR-205 caused by oxidative and ER stresses was evaluated by a loss- or gain-of-function study. Transfection of a miR-205 inhibitor decreased miR-205 expression by 0.005-fold in HK-2 (Figure 5A). The miR-205 suppression was associated with a slow growth as compared to the cells transfected with the control inhibitor (Figure 5B). The cell morphology was not obviously changed by transfection with miR-205 inhibitor compared to cells without transfection or to that on transfection with the control inhibitor (Figure 5C). The viability of cells treated with miR-205 inhibitor or control inhibitor without stimulation was 100% for each group (Figure 5E), showing that the difference in cell number was not due to cell injury induced by miR-205 inhibitor, but rather to repression of cell growth. The result of LDH assay also revealed that modulation of miR-205 expression did not affect cell death (Figure 5D). Importantly, these cells were more sensitive to oxidative or ER stress, with trypan blue exclusion assay showing a marked decrease in survival under either oxidative or ER stress conditions (Figure 5E). On the other hand, transfection of a miR-205 mimic by itself made no change in cell number and cell morphology (Figure 5B). In contrast, miR-205 overexpressing cells were more resistant to oxidative or ER stress, with trypan blue exclusion assay showing a significant increase in survival under either oxidative or ER stress conditions (Figure 5F). These results revealed that miR-205 in HK-2 modulated cell survival under both oxidative stress and ER stress.

**Figure 10:**
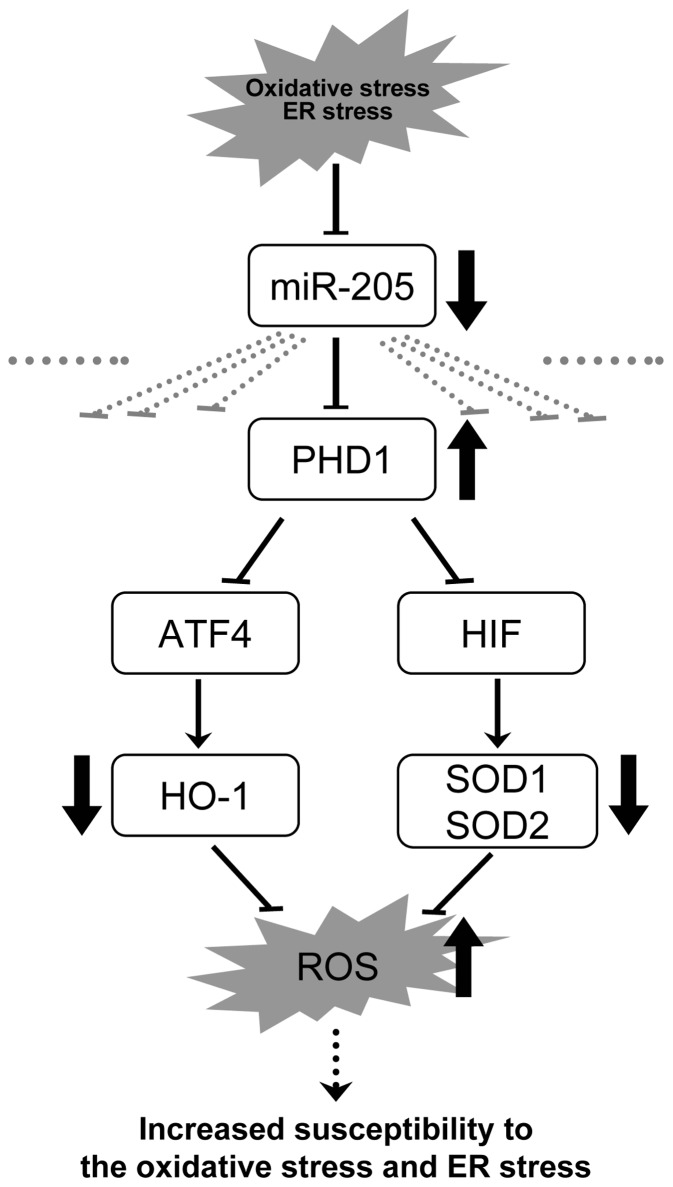
miR-205 contributes to both signalings triggered by oxidative and ER stresses in renal tubular cells. When cells are exposed to oxidative stress or ER stress, maladaptive down-regulation of miR-205 and subsequent up-regulation of EGLN2 occurs. ATF4 and HIF, which are negatively regulated by EGLN2, are supposed to contribute to the downstream suppression of anti-oxidant enzymes.

### Down-regulation of miR-205 Aggravated Oxidative Stress State

To investigate the mechanism of the change in cell survival by miR-205, we assessed the change in intracellular ROS level by FACS analysis utilizing CM-H2DCFDA. Of note, HK-2 with miR-205 inhibition showed an increase in basal intracellular ROS level as compared to control cells (Figure 6A, 6B). The increase in intracellular ROS level by H_2_O_2_ was significantly greater in miR205-inhibited HK-2 than control cells (Figure 6A, 6B). An increase in basal intracellular ROS level in miR-205-inhibited HK-2 was also confirmed by Western blot analysis of CEL, a major advanced glycation endproduct (AGE) and oxidative stress marker, which is known to be associated with various diseases, including renal diseases, caused by either or both chronic and acute oxidative stress [29], [30]: the intensity of bands with a molecular weight corresponding to 32, 23 and 17 kDa were increased in miR-205-inhibited HK-2 compared with control (Figure 6C). Because glycation is occurred in various proteins to form CEL, Western blot analysis of CEL reveals multiple bands. Thus, the repression of cell viability by miR-205 inhibition was associated with an increase in intracellular ROS level.

### miR-205 Altered Anti-oxidant Enzyme Expression

To investigate whether the increase in intracellular ROS level by miR-205 down-regulation was mediated by a change in anti-oxidant enzyme expression, we evaluated changes in the expression of these enzymes in HK-2 with miR-205 inhibition. Results consistently showed that the inhibition of miR-205 was associated with a decrease in the expression of anti-oxidant enzymes, such as copper/zinc superoxide dismutase (SOD1), manganese superoxide dismutase (SOD2), catalase, and hemoxygenase 1 (HO-1) (fold change compared to control of 0.63±0.06 in SOD1 (P<0.01), 0.66±0.07 in SOD2 (P<0.01), 0.58±0.06 in catalase (P<0.05), and 0.62±0.15 in HO-1 (P<0.01)) (Figure 7A). We also confirmed by Western blot analysis that SOD1 and HO-1 were significantly decreased at protein level in miR-205-inhibited HK-2 (Figure 7B, 7C).

While up-regulation of miR-205 *per se* made no change in the expression levels of these anti-oxidant enzymes (Figure 7A), miR205-overexpressing HK-2 cells showed increases in these anti-oxidant enzymes when exposed to oxidative or ER stress (Figure 7D–7G). On the contrary, down-regulation of miR-205 suppressed the stress induced-increase of these anti-oxidant enzymes (Figure 7D–7G). These results revealed that modulation of miR-205 altered the expression of anti-oxidant enzymes, resulting in the impairment of cell survival. These results also suggested that the basal expression level of miR-205 in HK-2 was sufficient to regulate intracellular ROS level, so that the effect of miR-205 overexpression via increase of anti-oxidant enzymes were exerted only when cells were under the stress conditions, namely when cells must be strongly protected against free radical damage by scavenging excessive ROS.

### EGLN2 was a Novel Target Gene of miR-205

Down-regulation of miR-205 reduced anti-oxidant enzyme expression, suggesting that miR-205 enhances anti-oxidant enzyme expression. Because miRNAs generally work as inhibitors of target genes, there should be a direct target of miR-205 that decreases anti-oxidant enzyme expression. To investigate target genes of miR-205 in the modulation of cell survival under stress conditions, we evaluated changes in the expression of stress signal regulators at the posttranscriptional level, which are related to mitochondrial oxidative stress pathways, hypoxia-inducible factor (HIF) pathways, and adaptive or proapoptotic UPR pathways, in HK-2 with knockdown of miR-205. Among them, EGLN2 was significantly increased by miR-205 inhibition, suggesting the suppressive regulation of EGLN2 by miR-205 (fold change compared to control was 1.77±0.24 in miR-205 knockdown, P<0.05) (Figure 8A). Western blot analysis of EGLN2 revealed that EGLN2 was also increased at protein level in miR-205-inhibited HK-2 (Figure 8B). The significant increases of EGLN2 mRNA expression were also observed in miR-205-inhibited HK-2 exposed to oxidative or ER stress (Figure 8C). These stress-responsive increases of EGLN2 were completely repressed by overexpression of miR-205 (Figure 8C), though EGLN2 was not significantly decreased by miR-205 overexpression without any stimulations (Figure 8A and 8C). In contrast, we found no change in the expression levels of EGLN1 (PHD2) or EGLN3 (PHD3) by both miR-205 modulations (Figure 8A).

*In silico* analysis revealed that the 3′- UTR of EGLN2 (transcript variant 1) has a putative binding site for miR-205 which contains its seed sequence (Figure 8D). We therefore performed luciferase assays of the wild or mutant EGLN2 3′-UTR (Figure 8E) after co-transfection with miR-205 mimic, inhibitor, or negative control. Transient transfection of HK-2 with EGLN2 3′-UTR-fLUC plasmids along with miR-205 inhibition led to an increase in relative luciferase activity compared with cells co-transfected with EGLN2 3′-UTR-fLUC plasmids and negative control (Figure 8F). This increase was not observed when mutant EGLN2 3′-UTR vector was co-transfected with miR-205 inhibitor (Figure 8F). The transfection of HK-2 with EGLN2 3′-UTR vector along with miR-205 overexpression did not lead significant decrease in luciferase activity compared with control (Figure 8F).

A relative luciferase activities were also significantly increased when transiently transfected HK-2 with EGLN2 3′-UTR fLUC plasmids were exposed to oxidative or ER stress, and these increases were canceled when mutant EGLN2 3′-UTR vectors were used (Figure 8G). Furthermore, co-transfection of EGLN2 3′-UTR fLUC plasmids with miR-205 mimic partially restrained these stress-induced increases of relative luciferase activities (Figure 8G). These results indicated that EGLN2 was a novel direct target of miR-205 in HK-2, and that the change of cell susceptibility to oxidative and ER stress by miR-205 modulation in HK-2 was mediated via EGLN2.

### EGLN2 Negatively Controlled HIF and ATF4 in HK-2

Because of that PHD is a crucial regulator of the stability of HIF, we evaluated changes in the expression of downstream targets of HIF, such as vascular endothelial growth factor (VEGF) and glucose transporter 1 (GLUT1), in HK-2 with miR-205 inhibition or overexpression. Results of real time RT-qPCR showed that the expression level of both VEGF and GLUT1 were decreased by miR-205 down-regulation, but not significantly changed by miR-205 overexpression (Figure 9A). To confirm these results, we arranged HRE-luciferase assay to assess the transcriptional activity of HIF-α, and the HRE-driven relative luciferase activity was significantly decreased by miR-205 inhibition (Figure 9B). Western blot analysis also revealed that both HIF1-α and HIF2-α were significantly decreased in miR-205-inhibited HK-2 (Figure 9C and 9D).

Meanwhile, recent study has demonstrated a novel function of EGLN2 whereby it directly represses the transcriptional activity of activating transcription factor 4 (ATF4), which is a crucial regulator of ER stress [20]. We thus performed Western blot analysis of ATF4, and the result showed a significant decrease in ATF4 by miR-205 inhibition (Figure 9E). These results indicated that EGLN2 was functionally regulated by miR-205, and that HIF and ATF4 indirectly contributed to the change of cell susceptibility to both oxidative and ER stresses induced by miR-205 modulation.

## Discussion

In this study, we used miRNA microarray analysis and quantitative real-time RT-PCR to examine changes in miRNA expression profiles in the human proximal tubular cell line HK-2 under hypoxia-reoxygenation-induced oxidative stress or ER stress conditions. Results showed that these stresses significantly changed the expression level of certain miRNAs. These included miR-210, which is known to be a direct transcriptional target of HIF-1α [24] and regulator of mitochondrial ROS production [25], [31]. miR29a, which acts as a repressor of collagen expression in tubular cells [27], [32], was also included. These findings suggest that the biological phenotypic changes in proximal tubular cells by oxidative or ER stress are mediated by miRNA expression, at least in part.

Further, we also identified miR-205 as an miRNA that is down-regulated in both oxidative and ER stress conditions. Recent studies have demonstrated that miR-205 has an epithelial-specific expression pattern with potential roles in both normal development and cancer [33], [34]. Potential targets of miR-205 in cancer proposed to date include tumor suppressors [34]–[36], oncogenes [37]–[41] and pro-metastatic genes [42], [43]. The function of miR-205 in cancer as either a tumor suppressor or oncogene thus depends on the type of tumor cell in which it is expressed. While previous studies of miR-205 focused on cancer, more recent studies have reported that expression is increased in kidney biopsy samples from patients with two non-tumor kidney diseases, namely hypertensive glomerulosclerosis or IgA nephropathy, and that the degree of upregulation is correlated with disease severity and progression [28], [44]. While these findings suggest that miR-205 plays a functionally important role in kidney disease, its effect in renal tubular cells has remained unclear. In our study, we demonstrated for the first time the effect of miR-205 on the survival of tubular cells exposed to stress conditions. miR-205 expression was significantly decreased in HK-2 under oxidative stress or ER stress, and cell susceptibility to these stresses was increased by down-regulation of miR-205, while cells became more resistant to these stresses by up-regulation of miR-205. These findings demonstrate that miR-205 protects proximal tubular cells against these stresses. Moreover, inhibition of miR-205 led to a decrease in cell number independently of cell death, suggesting that the damage of miR-205 inhibition was not lethal for HK-2 but severe enough to suppress cell growth. Though the mechanisms for cell growth suppression in miR-205-inhibited HK-2 remain elusive, recent evidences that miR-205 regulates the expression of several tumor-suppressor proteins and increases cellular proliferation in non-cancerous cells [34]–[36] might be supportive for our results.

We identified EGLN2 as a novel target of miR-205 in tubular cells by 3′-UTR-luciferase assay, Western blot analysis, and quantitative real-time RT-PCR. For targeting of 3′-UTRs by miRNAs, miRNA seed sequence has to be complementary to their targets. In contrast, the complementarity of the seed region of miR-205 to EGLN2 3′-UTR is relatively lower than that the previous paper suggested. In other target gene of miR-205 in renal cancer cells, it was also demonstrated the similar binding pattern in targeting of LYN 3′-UTR by miR-205 [40]. It may suggest the possibility that targeting of 3′-UTRs by miRNAs depend on not only seed sequence but also its adjacent sequence.

We found that miR-205 regulates intracellular ROS levels via the alteration of anti-oxidant enzymes, such as SOD1, SOD2, catalase, and HO-1. How does down-regulation of EGLN2 by miR-205 protect cells via the modulation of anti-oxidant enzymes and intracellular ROS? While PHD is a crucial regulator of the stability of HIF, the master gene in the adaptive responses against hypoxia, metabolic analysis of EGLN2 knockout mice revealed that EGLN2 deficiency ameliorates oxidative stress in a HIF-2α-dependent manner, which subsequently preserves mitochondrial respiration and provides sufficient ATP for survival [45]. We have also previously shown that HIF-2α knockdown mice have lower baseline expression levels of anti-oxidant enzymes in the kidney, such as SOD1, SOD2, and GPX1, and that these mice exhibit severe histological changes on ischemia-reperfusion injury [46]. Moreover recent study has demonstrated that selective inhibition of EGLN2 but not EGLN1 or EGLN3 prevents normoxic oxidative neuronal injury in a HIF-2α-dependent manner [47]. We therefore speculate that a decrease in miR-205 leads to the up-regulation of EGLN2 and subsequent down-regulation of HIF-2α, resulting in excess generation of ROS within mitochondria and the suppression of anti-oxidant enzymes, which in turn increase susceptibility to oxidative stress in tubular cells. In fact, our results of HRE-luciferase assays and Western blot analysis of HIF-1α and HIF-2α supported this speculation. On this basis, we suggest that down-regulation of miR-205 with subsequent up-regulation of EGLN2 serves as a maladaptive response, which aggravates cell injury due to oxidative stress in reoxygenation.

Our current study demonstrated that miR-205 is a miRNA that is down-regulated in both ER stress and oxidative stress conditions. We also found that PHD regulates ER stress responses. Interestingly, inhibition of PHD increases expression of oxygen-regulated protein 150 (ORP150), a representative ER stress-inducible chaperone, in COS-7 cells [48], and activates all three regulatory arms of the UPR in murine microvascular endothelial cells and in mouse hearts, which serves as a protective mechanism against hypoxia/oxidative stress-induced ER stress [49]. The defensive mechanism of the inhibition of PHD includes the induction of pro-survival anti-oxidants. On the other hand, a recent study has demonstrated a novel function of EGLN2 whereby it directly represses the transcriptional activity of activating transcription factor 4 (ATF4), which is a crucial regulator of ER stress, without prolyl hydroxylation [20]. Target genes of ATF4 include genes for redox balance, such as HO-1, which mediates an adaptive response to oxidative stress [50]. Thus, ATF4 may be the key protein in the signaling triggered by both oxidative and ER stresses in a miR-205-dependent manner. In the present study, we showed that the inhibition of miR-205 indeed induced the downregulation of ATF4, and HO-1 was also decreased both in mRNA and protein level, suggesting that ATF4 may play a role in cell preservation against oxidative and ER stresses in cooperation with EGLN2. On this basis, the down-regulation of miR-205 with subsequent up-regulation of EGLN2 would again be maladaptive, exacerbating cell damage due to ER stress.

In contrast, up-regulation of miR-205 by itself did not significantly change the expression of EGLN2, and neither did anti-oxidant enzymes under physiological conditions. The cell protective effect of miR-205 in cooperation with EGLN2 were exerted only when miR-205 overexpressed HK-2 were exposed to oxidative or ER stress, suggesting that the basal expression level of miR-205 in HK-2 was sufficient for this cell protective cascade. Moreover, because prolyl hydroxylases are crucial for cell survival, miR-205 must not be the only regulator of EGLN2. Thus, the effects of up-regulation of miR-205 with subsequent down-regulation of EGLN2 might be counteracted to some extent by other regulators of EGLN2 at least under non-stimulus condition.

The current study showed that the expression of pre-miR-205 as well as mature miR-205 was altered by oxidative and ER stresses, indicating the possibility of that transcription of miR-205 was inhibited by certain pathogenic factor(s). These data emphasize the pathophysiological significance of regulation of miR-205 expression. The detailed mechanism of transcriptional regulation and processing capacity of miR-205 under oxidative and ER stresses should be elucidated.

We conclude that miR-205 serves a protective role against both oxidative stress and ER stress. However, when cells are exposed to oxidative stress or ER stress, maladaptive down-regulation of miR-205 and subsequent up-regulation of EGLN2 occurs. EGLN2 is one of the novel direct targets of miR-205, and its up-regulation by inhibiting miR-205 leads to subsequent down-regulation of HIF and ATF4, then finally increases the intracellular ROS via suppression of HIF/ATF4-regulated anti-oxidant enzymes, and renders the cells more vulnerable to these stresses (Figure 10).

## References

[1] Kim JW, Tchernyshyov I, Semenza GL, Dang CV (2006) HIF-1-mediated expression of pyruvate dehydrogenase kinase: a metabolic switch required for cellular adaptation to hypoxia. Cell Metab 3: 177–185. 16517405 10.1016/j.cmet.2006.02.002

[2] Papandreou I, Cairns RA, Fontana L, Lim AL, Denko NC (2006) HIF-1 mediates adaptation to hypoxia by actively downregulating mitochondrial oxygen consumption. Cell Metab 3: 187–197. 16517406 10.1016/j.cmet.2006.01.012

[3] Nangaku M (2006) Chronic hypoxia and tubulointerstitial injury: a final common pathway to end-stage renal failure. J Am Soc Nephrol 17: 17–25. 16291837 10.1681/ASN.2005070757

[4] Mimura I, Nangaku M (2010) The suffocating kidney: tubulointerstitial hypoxia in end-stage renal disease. Nat Rev Nephrol 6: 667–678. 20877304 10.1038/nrneph.2010.124

[5] Inagi R (2010) Endoplasmic reticulum stress as a progression factor for kidney injury. Curr Opin Pharmacol 10: 156–165. 20045381 10.1016/j.coph.2009.11.006

[6] Cybulsky AV, Takano T, Papillon J, Kitzler TM, Bijian K (2011) Endoplasmic reticulum stress in glomerular epithelial cell injury. Am J Physiol Renal Physiol 301: F496–508. 21159733 10.1152/ajprenal.00728.2010

[7] Cunard R, Sharma K (2011) The endoplasmic reticulum stress response and diabetic kidney disease. Am J Physiol Renal Physiol 300: F1054–1061. 21345978 10.1152/ajprenal.00021.2011 PMC3094049

[8] Cybulsky AV (2010) Endoplasmic reticulum stress in proteinuric kidney disease. Kidney Int 77: 187–193. 19812538 10.1038/ki.2009.389

[9] Kawakami T, Inagi R, Wada T, Tanaka T, Fujita T, et al (2010) Indoxyl sulfate inhibits proliferation of human proximal tubular cells via endoplasmic reticulum stress. Am J Physiol Renal Physiol 299: F568–576. 20534867 10.1152/ajprenal.00659.2009

[10] Inagi R, Kumagai T, Nishi H, Kawakami T, Miyata T, et al (2008) Preconditioning with endoplasmic reticulum stress ameliorates mesangioproliferative glomerulonephritis. J Am Soc Nephrol 19: 915–922. 18256359 10.1681/ASN.2007070745 PMC2386726

[11] Ohse T, Inagi R, Tanaka T, Ota T, Miyata T, et al (2006) Albumin induces endoplasmic reticulum stress and apoptosis in renal proximal tubular cells. Kidney Int 70: 1447–1455. 16955111 10.1038/sj.ki.5001704

[12] Inagi R (2009) Endoplasmic reticulum stress in the kidney as a novel mediator of kidney injury. Nephron Exp Nephrol 112: e1–9. 19342868 10.1159/000210573

[13] Bando Y, Tsukamoto Y, Katayama T, Ozawa K, Kitao Y, et al (2004) ORP150/HSP12A protects renal tubular epithelium from ischemia-induced cell death. FASEB J 18: 1401–1403. 15240565 10.1096/fj.03-1161fje

[14] Hayashi T, Saito A, Okuno S, Ferrand-Drake M, Dodd RL, et al (2003) Oxidative damage to the endoplasmic reticulum is implicated in ischemic neuronal cell death. J Cereb Blood Flow Metab 23: 1117–1128. 14526222 10.1097/01.WCB.0000089600.87125.AD

[15] Yokouchi M, Hiramatsu N, Hayakawa K, Okamura M, Du S, et al (2008) Involvement of selective reactive oxygen species upstream of proapoptotic branches of unfolded protein response. J Biol Chem 283: 4252–4260. 18086661 10.1074/jbc.M705951200

[16] Filipowicz W, Bhattacharyya SN, Sonenberg N (2008) Mechanisms of post-transcriptional regulation by microRNAs: are the answers in sight? Nat Rev Genet 9: 102–114. 18197166 10.1038/nrg2290

[17] Bartel DP (2004) MicroRNAs: genomics, biogenesis, mechanism, and function. Cell 116: 281–297. 14744438 10.1016/s0092-8674(04)00045-5

[18] Lee Y, Ahn C, Han J, Choi H, Kim J, et al (2003) The nuclear RNase III Drosha initiates microRNA processing. Nature 425: 415–419. 14508493 10.1038/nature01957

[19] Lorenzen JM, Haller H, Thum T (2011) MicroRNAs as mediators and therapeutic targets in chronic kidney disease. Nat Rev Nephrol 7: 286–294. 21423249 10.1038/nrneph.2011.26

[20] Hiwatashi Y, Kanno K, Takasaki C, Goryo K, Sato T, et al (2011) PHD1 interacts with ATF4 and negatively regulates its transcriptional activity without prolyl hydroxylation. Exp Cell Res. 10.1016/j.yexcr.2011.09.005 21951999

[21] Ryan MJ, Johnson G, Kirk J, Fuerstenberg SM, Zager RA, et al (1994) HK-2: an immortalized proximal tubule epithelial cell line from normal adult human kidney. Kidney Int 45: 48–57. 8127021 10.1038/ki.1994.6

[22] Tsai CA, Chen YJ, Chen JJ (2003) Testing for differentially expressed genes with microarray data. Nucleic Acids Res 31: e52. 12711697 10.1093/nar/gng052 PMC154240

[23] Tanaka T, Miyata T, Inagi R, Fujita T, Nangaku M (2004) Hypoxia in renal disease with proteinuria and/or glomerular hypertension. American Journal of Pathology 165: 1979–1992. 15579441 10.1016/S0002-9440(10)63249-X PMC1618699

[24] Huang X, Ding L, Bennewith KL, Tong RT, Welford SM, et al (2009) Hypoxia-inducible mir-210 regulates normoxic gene expression involved in tumor initiation. Mol Cell 35: 856–867. 19782034 10.1016/j.molcel.2009.09.006 PMC2782615

[25] Mutharasan RK, Nagpal V, Ichikawa Y, Ardehali H (2011) microRNA-210 is Upregulated in Hypoxic Cardiomyocytes through Akt- and p53-dependent Pathways and Exerts Cytoprotective Effects. Am J Physiol Heart Circ Physiol. 10.1152/ajpheart.01080.2010 PMC3197368 21841015

[26] Chen Z, Li Y, Zhang H, Huang P, Luthra R (2010) Hypoxia-regulated microRNA-210 modulates mitochondrial function and decreases ISCU and COX10 expression. Oncogene 29: 4362–4368. 20498629 10.1038/onc.2010.193

[27] Du B, Ma LM, Huang MB, Zhou H, Huang HL, et al (2010) High glucose down-regulates miR-29a to increase collagen IV production in HK-2 cells. FEBS Lett 584: 811–816. 20067797 10.1016/j.febslet.2009.12.053

[28] Wang G, Kwan BC, Lai FM, Choi PC, Chow KM, et al (2010) Intrarenal expression of miRNAs in patients with hypertensive nephrosclerosis. Am J Hypertens 23: 78–84. 19910931 10.1038/ajh.2009.208

[29] Kumagai T, Nangaku M, Kojima I, Nagai R, Ingelfinger JR, et al (2009) Glyoxalase I overexpression ameliorates renal ischemia-reperfusion injury in rats. Am J Physiol Renal Physiol 296: F912–921. 19211689 10.1152/ajprenal.90575.2008

[30] Beisswenger PJ, Drummond KS, Nelson RG, Howell SK, Szwergold BS, et al (2005) Susceptibility to diabetic nephropathy is related to dicarbonyl and oxidative stress. Diabetes 54: 3274–3281. 16249455 10.2337/diabetes.54.11.3274

[31] Chan SY, Zhang YY, Hemann C, Mahoney CE, Zweier JL, et al (2009) MicroRNA-210 controls mitochondrial metabolism during hypoxia by repressing the iron-sulfur cluster assembly proteins ISCU1/2. Cell Metab 10: 273–284. 19808020 10.1016/j.cmet.2009.08.015 PMC2759401

[32] Wang B, Komers R, Carew R, Winbanks CE, Xu B, et al (2012) Suppression of microRNA-29 expression by TGF-beta1 promotes collagen expression and renal fibrosis. Journal of the American Society of Nephrology 23: 252–265. 22095944 10.1681/ASN.2011010055 PMC3269175

[33] Greene SB, Herschkowitz JI, Rosen JM (2010) The ups and downs of miR-205: identifying the roles of miR-205 in mammary gland development and breast cancer. RNA Biol 7: 300–304. 20436283 10.4161/rna.7.3.11837 PMC3008649

[34] Greene SB, Gunaratne PH, Hammond SM, Rosen JM (2010) A putative role for microRNA-205 in mammary epithelial cell progenitors. J Cell Sci 123: 606–618. 20103531 10.1242/jcs.056812 PMC2818197

[35] Yu J, Ryan DG, Getsios S, Oliveira-Fernandes M, Fatima A, et al (2008) MicroRNA-184 antagonizes microRNA-205 to maintain SHIP2 levels in epithelia. Proc Natl Acad Sci U S A 105: 19300–19305. 19033458 10.1073/pnas.0803992105 PMC2587229

[36] Yu J, Peng H, Ruan Q, Fatima A, Getsios S, et al (2010) MicroRNA-205 promotes keratinocyte migration via the lipid phosphatase SHIP2. Faseb J 24: 3950–3959. 20530248 10.1096/fj.10-157404 PMC2996908

[37] Iorio MV, Casalini P, Piovan C, Di Leva G, Merlo A, et al (2009) microRNA-205 regulates HER3 in human breast cancer. Cancer Res 69: 2195–2200. 19276373 10.1158/0008-5472.CAN-08-2920

[38] Dar AA, Majid S, de Semir D, Nosrati M, Bezrookove V, et al (2011) miRNA-205 suppresses melanoma cell proliferation and induces senescence via regulation of E2F1 protein. J Biol Chem 286: 16606–16614. 21454583 10.1074/jbc.M111.227611 PMC3089503

[39] Gandellini P, Folini M, Longoni N, Pennati M, Binda M, et al (2009) miR-205 Exerts tumor-suppressive functions in human prostate through down-regulation of protein kinase Cepsilon. Cancer Res 69: 2287–2295. 19244118 10.1158/0008-5472.CAN-08-2894

[40] Majid S, Saini S, Dar AA, Hirata H, Shahryari V, et al (2011) MicroRNA-205 inhibits Src-mediated oncogenic pathways in renal cancer. Cancer Res 71: 2611–2621. 21330408 10.1158/0008-5472.CAN-10-3666 PMC3940352

[41] Piovan C, Palmieri D, Di Leva G, Braccioli L, Casalini P, et al (2012) Oncosuppressive role of p53-induced miR-205 in triple negative breast cancer. Mol Oncol. 10.1016/j.molonc.2012.03.003 PMC3679926 22578566

[42] Gregory PA, Bert AG, Paterson EL, Barry SC, Tsykin A, et al (2008) The miR-200 family and miR-205 regulate epithelial to mesenchymal transition by targeting ZEB1 and SIP1. Nat Cell Biol 10: 593–601. 18376396 10.1038/ncb1722

[43] Matsushima K, Isomoto H, Yamaguchi N, Inoue N, Machida H, et al (2011) MiRNA-205 modulates cellular invasion and migration via regulating zinc finger E-box binding homeobox 2 expression in esophageal squamous cell carcinoma cells. J Transl Med 9: 30. 21426561 10.1186/1479-5876-9-30 PMC3076245

[44] Wang G, Kwan BC, Lai FM, Choi PC, Chow KM, et al (2010) Intrarenal expression of microRNAs in patients with IgA nephropathy. Lab Invest 90: 98–103. 19901913 10.1038/labinvest.2009.118

[45] Aragones J, Schneider M, Van Geyte K, Fraisl P, Dresselaers T, et al (2008) Deficiency or inhibition of oxygen sensor Phd1 induces hypoxia tolerance by reprogramming basal metabolism. Nat Genet 40: 170–180. 18176562 10.1038/ng.2007.62

[46] Kojima I, Tanaka T, Inagi R, Kato H, Yamashita T, et al (2007) Protective role of hypoxia-inducible factor-2alpha against ischemic damage and oxidative stress in the kidney. J Am Soc Nephrol 18: 1218–1226. 17344427 10.1681/ASN.2006060639

[47] Siddiq A, Aminova LR, Troy CM, Suh K, Messer Z, et al (2009) Selective inhibition of hypoxia-inducible factor (HIF) prolyl-hydroxylase 1 mediates neuroprotection against normoxic oxidative death via HIF- and CREB-independent pathways. Journal of Neuroscience 29: 8828–8838. 19587290 10.1523/JNEUROSCI.1779-09.2009 PMC3290095

[48] Arrington DD, Schnellmann RG (2008) Targeting of the molecular chaperone oxygen-regulated protein 150 (ORP150) to mitochondria and its induction by cellular stress. Am J Physiol Cell Physiol 294: C641–650. 18094145 10.1152/ajpcell.00400.2007

[49] Natarajan R, Salloum FN, Fisher BJ, Smithson L, Almenara J, et al (2009) Prolyl hydroxylase inhibition attenuates post-ischemic cardiac injury via induction of endoplasmic reticulum stress genes. Vascul Pharmacol 51: 110–118. 19524066 10.1016/j.vph.2009.05.007

[50] He CH, Gong P, Hu B, Stewart D, Choi ME, et al (2001) Identification of activating transcription factor 4 (ATF4) as an Nrf2-interacting protein. Implication for heme oxygenase-1 gene regulation. J Biol Chem 276: 20858–20865. 11274184 10.1074/jbc.M101198200

